# Endothelial Dysfunction May Link Interatrial Septal Abnormalities and MTHFR-Inherited Defects to Cryptogenic Stroke Predisposition

**DOI:** 10.3390/biom10060861

**Published:** 2020-06-04

**Authors:** Luca Sgarra, Alessandro Santo Bortone, Maria Assunta Potenza, Carmela Nacci, Maria Antonietta De Salvia, Tommaso Acquaviva, Emanuela De Cillis, Marco Matteo Ciccone, Massimo Grimaldi, Monica Montagnani

**Affiliations:** 1 Department of Biomedical Sciences and Human Oncology—Section of Pharmacology, Medical School, University of Bari “Aldo Moro”, 70124 Bari, Italy; sgarraluca@gmail.com (L.S.); mariaassunta.potenza@uniba.it (M.A.P.); carmela.nacci@uniba.it (C.N.); mariaantonietta.desalvia@uniba.it (M.A.D.S.); 2 Department of Emergency and Organ Transplantation—Section of Cardiovascular Diseases, Medical School, University of Bari “Aldo Moro”, 70124 Bari, Italy; alessandrosanto.bortone@uniba.it (A.S.B.); tommasoacquaviva68@libero.it (T.A.); emanuela.decillis@gmail.com (E.D.C.); marcomatteo.ciccone@uniba.it (M.M.C.); 3 General Hospital “F. Miulli” Acquaviva delle Fonti, 70021 Bari, Italy; fiatric@hotmail.com

**Keywords:** endothelial dysfunction, L-Arg/ADMA, PFO, MTHFR, cryptogenic stroke

## Abstract

We explored the significance of the L-Arginine/asymmetric dimethylarginine (L-Arg/ADMA) ratio as a biomarker of endothelial dysfunction in stroke patients. To this aim, we evaluated the correlation, in terms of severity, between the degree of endothelial dysfunction (by L-Arg/ADMA ratio), the methylene tetrahydrofolate reductase (MTHFR) genotype, and the interatrial septum (IAS) phenotype in subject with a history of stroke. **Methods and Results:** L-Arg, ADMA, and MTHFR genotypes were evaluated; the IAS phenotype was assessed by transesophageal echocardiography. Patients were grouped according to the severity of IAS defects and the residual enzymatic activity of MTHFR-mutated variants, and values of L-Arg/ADMA ratio were measured in each subgroup. Of 57 patients, 10 had a septum integrum (SI), 38 a patent foramen ovale (PFO), and 9 an ostium secundum (OS). The L-Arg/ADMA ratio differed across septum phenotypes (*p* ≤ 0.01), and was higher in SI than in PFO or OS patients (*p* ≤ 0.05, *p* ≤ 0.01, respectively). In the PFO subgroup a negative correlation was found between the L-Arg/ADMA ratio and PFO tunnel length/height ratio (*p* ≤ 0.05; r = − 0.37; R2 = 0.14). Interestingly, the L-Arg/ADMA ratio varied across MTHFR genotypes (*p* ≤ 0.0001) and was lower in subgroups carrying the most impaired enzyme with respect to patients carrying the conservative MTHFR (*p* ≤ 0.0001, *p* ≤ 0.05, respectively). Consistently, OS patients carried the most dysfunctional MTHFR genotypes, whereas SI patients the least ones. **Conclusions:** A low L-Arg/ADMA ratio correlates with impaired activity of MTHFR and with the jeopardized IAS phenotype along a severity spectrum encompassing OS, PFO with long/tight tunnel, PFO with short/large tunnel, and SI. This infers that genetic MTHFR defects may underlie endothelial dysfunction-related IAS abnormalities, and predispose to a cryptogenic stroke. Our findings emphasize the role of the L-Arg/ADMA ratio as a reliable marker of stroke susceptibility in carriers of IAS abnormalities, and suggest its potential use both as a diagnostic tool and as a decision aid for therapy.

## 1. Introduction

The search for causes and mechanisms underlying strokes is particularly important in young patients, where the absence of significant small- and large-vessel disease, and/or dissection accounts for the higher number of strokes diagnosed as cryptogenic [1]. The role of interatrial septum (IAS) defect features [2,3] in the so-called paradoxical embolism is currently investigated. Patent foramen ovale (PFO) is a frequent IAS abnormality, and the potential advantages in secondary prevention of surgical closure over medical therapy are unclear [4,5,6,7,8,9]. Echocardiographic assessment of PFO interatrial tunnel length has produced conflicting results as well, initially suggesting a greater risk to cryptogenic stroke in patients with larger defects [10], and recently re-evaluating such a statement on the basis of RoPE database analysis [11]. Similarly, the correlative risk of embolism and stroke based on interatrial shunt extent has generated inconclusive, if not controversial, indications [12,13]. In this scenario of diagnostic and therapeutic uncertainty, additional evidence for risk stratification is highly pursued, and the search for potential biomarkers is strongly encouraged.

A position paper from the Italian SICI-GISE Society suggests that thrombophilia is an additional risk factor for stroke predisposition, sufficient to replace pharmacological therapy with PFO percutaneous surgery [14]. Indeed, PFO closure is more effective than medical therapy to mitigate stroke recurrence in thrombophilic subjects [15]; similarly, PT_G20210A_ and FV_G1691A_ mutations are more frequently associated in patients with PFO-related cerebral infarcts than in a control population [16,17]. In the potential relationship linking thrombophilia to cryptogenic stroke, the predisposition conferred by genetic defects in the methylene tetrahydrofolate reductase (MTHFR) is one under-investigated condition.

MTHFR is a key enzyme in the folate cycle, whose vitamin B12-dependent conversion of homocysteine to methionine produces the main cellular carrier for methylation [18]. Polymorphisms of the MTHFR include the 677 C > T and the 1298 A > C substitutions, associated to a progressive loss of enzymatic activity from A1298C heterozygosity to C677T homozygosity [19,20]. Defective MTHFR genes increase homocysteine levels, and hyperhomocysteinemia has been associated to higher risk for atherosclerosis, cardiovascular events, venous thrombosis, and microangiopathy [21]. Similarly, MTHFR C677T and A1298C polymorphisms have been associated with multiple small-artery occlusion [22], a subcortical pattern of potential embolic origin [23,24], and with stroke in patients with large-vessel disease [25].

While sharing stroke aptitude and epidemiology, MTHFR polymorphisms and PFO are regarded as unrelated conditions that can overlap rather than interact. However, it is noteworthy that PFO carriers show higher plasma homocysteine levels than patients without PFO [26]. Thus, from the observations described above, genetic MTHFR defects may lie beneath both inherited thrombophilia and the IAS phenotype, and the missing link explaining both conditions might be endothelial dysfunction. We hypothesize that, by impairing endothelial activity, MTHFR malfunctioning may influence the physiological structure of IAS; in addition to disturbances in the coagulation process, this might represent one mechanism underlying nonobvious sources of cardiovascular embolism, and therefore help to explain the etiology of cryptogenic strokes.

To test this hypothesis, we evaluated the potential correlation between the severity of MTHFR mutation (considering both MTHFR polymorphism and homocysteinemia levels), the degree of endothelial dysfunction (indirectly measured as L-Arg/ADMA levels), and the IAS phenotype (by echocardiographic assessment) in patients diagnosed with Embolic Stroke of Undetemined Source (ESUS).If confirmed, this hypothesis might also help to legitimize L-Arg/ADMA as a marker of endothelial dysfunction.

## 2. Materials and Methods

### 2.1. Study Population and Enrolment Criteria

This retrospective observational study was carried in accordance with the guiding principles of the Declaration of Helsinki, with the approval of the local Ethical Committee on Human Research for non-interventional studies (Comitato Etico Indipendente, CE n. 4398, 03/12/2014). Fifty-seven subjects were consecutively enrolled among patients admitted between January 2017 and March 2019 to our Cardiology Unit with a diagnosis of ESUS [27]. Multiple and/or bilateral patterns were considered of embolic origin provided that, irrespective of its cerebral location, at least one lesion would be ≥ 15 mm, in the absence of a previous injury at the same site. All patients received standard, individually adjusted, cardiovascular therapy. In each patient, a 12-lead ECG, precordial and transesophageal echocardiography, Holter cardiac monitoring, MR and/or CT angiography of the brain ischemic area, and epiaortic and transcranial Doppler ultrasonography were performed. Patients were not included in the study if their age was ≥ 60 years, had a history of Congestive Heart Failure (CHF), atrial fibrillation (AF) or similar supraventricular arrhythmias, or left ventricular dysfunction, cancer, acute and/or chronic inflammatory disease, were on immunosuppressive therapy, or concomitantly taking vitamin or protein supplements. Each participant gave written informed consent before entering the study.

### 2.2. Laboratory Test

MTHFR polymorphisms were evaluated by RT-PCR on genomic DNA from peripheral blood samples according to standard protocols. Serum folate and vitamin B12 levels were measured by a chemiluminescent immunoassay (Tosoh Bioscience, AIA-PACK, Tessenderlo, Belgium). Homocysteine levels were measured by nephelometric analysis. Plasma asymmetric dimethylarginine (ADMA) and L-Arginine (L-Arg) levels were measured by enzyme-linked immunoassay (DLD Diagnostika GMBH, Hamburg, Germany) [28].

### 2.3. Echocardiographic Imaging

Echocardiographic analysis was performed (Philips EPIQ 7, Philips Healthcare, Andover, Philips S.p.A. Milan, Italy) using transducer frequencies of 5 to 7.5 MHz. Color Doppler mapping was set on Nyquist velocities from 28 to 55 cm/s during atrial septal examination. Transcranial and epiaortic Doppler sonography was aimed at ruling out concomitant stenosis/occlusion in the cerebral and/or precerebral vasculature, to ascertain the integrity of diastolic and systolic flow in the middle cerebral artery, and to detect and quantify the existence of shunts. Transthoracic echocardiography was aimed at ruling out valve calcification, myxomatous valvulopathies, aortic stenosis, mural and atrial thrombi, cardiac masses, ventricular dysfunction, ventricular non-compaction, endomyocardial fibrosis, endocarditic processes, and aortic arch atherosclerotic plaques, as well as to measure the size of the hearts’ right chambers. Transesophageal echocardiography was performed to confirm the existence of interatrial shunt, to detect and quantify shunt extent, and to provide morphologic features of PFO tunnel or OS defects [10]. In order to assess interobserver reproducibility of echographic measures, a second sonographer experienced in the field performed a second blind measurement of previously acquired images.

### 2.4. Statistical Analysis

All data were expressed as the mean ± error standard or the mean ± standard deviation, as indicated in the figures and tables. Kruskal–Wallis analysis of one-way ANOVA data was used to assess statistical significance across groups; the very same analysis followed by Dunn’s multiple comparisons correction was used for multiple testing. The correlation between the Arg/ADMA ratio and length/height of the PFO tunnel was assessed by the Pearson correlation coefficient. The Bland–Altman plots with 95% CIs for correlations were utilized to assess interobserver reproducibility of PFO tunnel morphology (Figure 4). Statistical significance (*p* ≤ 0.05) measured with GraphPad Prism 6.0 (GraphPad Inc., La Jolla, CA, USA) software is indicated in figure legends.

## 3. Results

### 3.1. Clinical and Laboratory Characteristics of Enrolled Patients

In 57 patients, a diagnosis of ESUS was made [27]. Baseline demographic, laboratory, and clinical characteristics of subjects are shown according to their IAS phenotype (Table 1) or MTHFR genotype (Table 2). The mean age of all participants was 41.2 years (range 27–57), with no significant difference among subgroups. Likewise, no difference in glucose levels, lipid profile, or blood pressure values were observed among patients (Table 1 and Table 2).

### 3.2. Morphologic Features of the IAS Defects and Folate-Related Metabolism

Out of 57 subjects, 10 had a septum integrum (SI, 17.5%), 38 carried a patency of foramen ovale (PFO, 66.7%), and 9 an ostium secundum defect (OS, 15.8%). For PFO, the average length/height tunnel was 10.6/3.47 mm, with a mean length/height ratio of 3.48 ± 0.22. For OS defect, the mean superior-inferior diameter was 16.5 mm and anterior-posterior diameter of 13.5 mm. Baseline characteristics of patients sub-grouped according to their IAS phenotype are shown in Table 1. Folic acid and homocysteine levels tended to be higher, whereas vitamin B12 tended to be lower among patients with PFO or OS than in SI patients (*p* = 0.049 and *p* = 0.039, respectively).

### 3.3. Distribution of MTHFR Genetic Variants and Folate-Related Metabolism

Sixteen participants carried the 677 T/T homozygous genotype (28.1%), 14 carried the 677 C/T + 1298 A/C double heterozygous mutation (24.6%), 6 carried a 677 C/T heterozygous genotype (10.5%), 5 were carriers of the homozygous 1298 C/C mutation (8.7%), and 16 were carriers of the 1298 A/C variant or wild-type MTHFR (28.1%). As shown in Table 2, levels of vitamin B12 and folic acid did not significantly differ among patients. Conversely, homocysteine levels were higher in the 677 T/T subgroup with respect to all other genotype subgroups (* *p* < 0.01).

### 3.4. L-Arg/ADMA Ratio Related to IAS Phenotype and MTHFR Genotype

**Figure 1:**
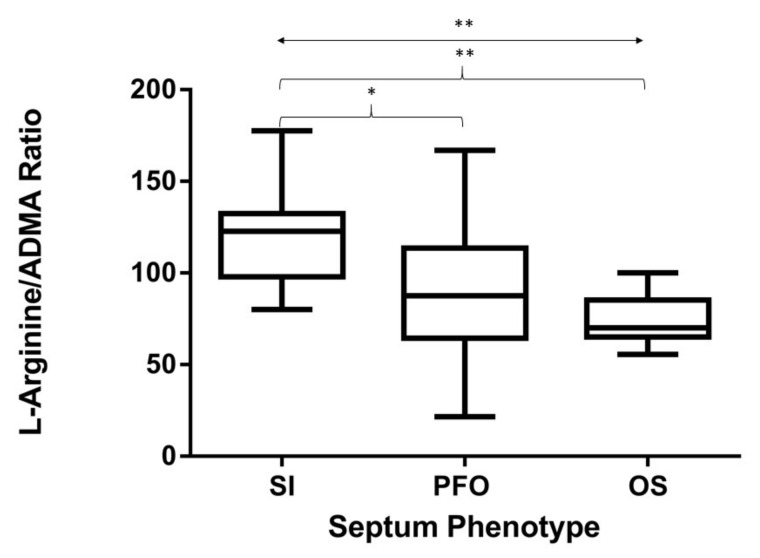
L-Arginine/asymmetric dimethylarginine (L-Arg/ADMA) ratios according to septum phenotype subclassification. Box plots indicate the median, maximum, and minimum values. The p-value across groups (double arrow line) was calculated by a Kruskall–Wallis non-parametric test of one-way ANOVA data. The p-value between groups (curly brackets) was calculated by Dunn’s multiple correction. * *p* ≤ 0.05; ** *p* ≤ 0.01.

The L-Arg/ADMA ratio significantly differed across groups (*p* < 0.01) and was significantly higher in SI patients (mean 119.3 ± 8.6) than in patients carrying a PFO (89 ± 5.3) and/or an OS defect (74.6 ± 4.6) (*p* ≤ 0.05 and *p* ≤ 0.01, respectively) (Figure 1).

**Figure 2:**
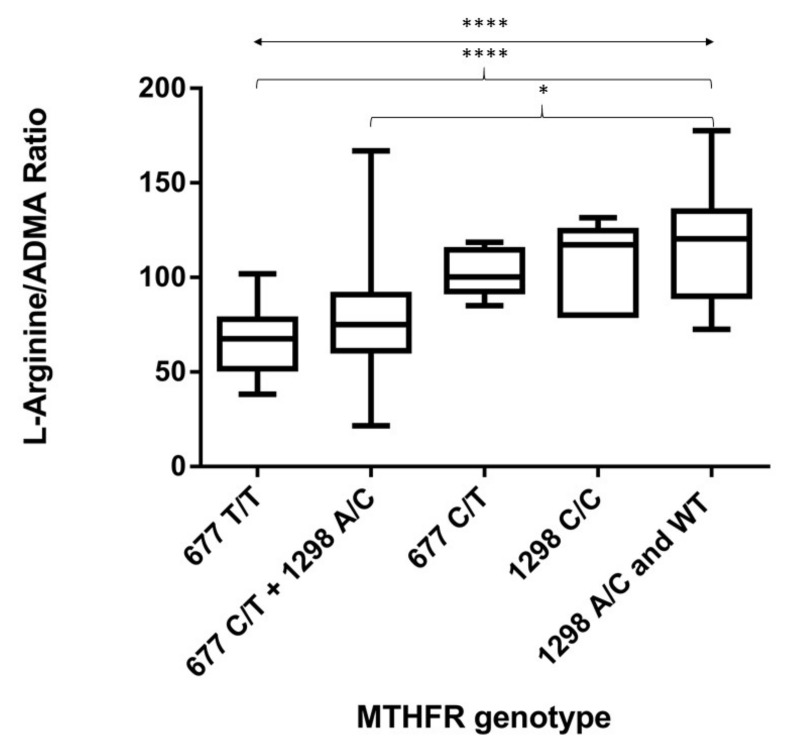
L-Arg/ADMA ratios according to MTHFR genotype subclassification. Box plots indicate the median, maximum, and minimum values. The p-value across groups (double arrow line) was calculated by a Kruskall–Wallis non-parametric test of one-way ANOVA data. The *p*-value between groups (curly brackets) was calculated by Dunn’s multiple correction. * *p* ≤ 0.05; **** *p* ≤ 0.0001.

The L-Arg/ADMA mean values were 67.5 ± 4.6 in patients of the MTHFR 677 T/T subgroup; 81 ± 9.4 in the 677 C/T + 1298 A/C subgroup; 102 ± 4.5 in the 677 C/T subgroup; 105.4 ± 9.5 in the 1298 C/C subgroup; and 116.4 ± 9.6 for patients of the 1298 A/C + WT subgroup. The L-Arg/ADMA ratio was significantly lower in both the most detrimental 677 T/T subgroup and in the 677 C/T + 1298 A/C heterozygous subgroup with respect to the healthiest 1298 A/C and WT subgroup (*p* ≤ 0.0001 and *p* ≤ 0.05, respectively) (Figure 2).

When combined (irrespective of MTHFR mutation or IAS phenotype), the mean L-Arg/ADMA ratio was lower in the whole group of our cryptogenic stroke patients than in healthy subjects, although still higher if compared to the L-Arg/ADMA ratio obtained in patients with acute myocardial infarction (Figure S1).

**Figure 3:**
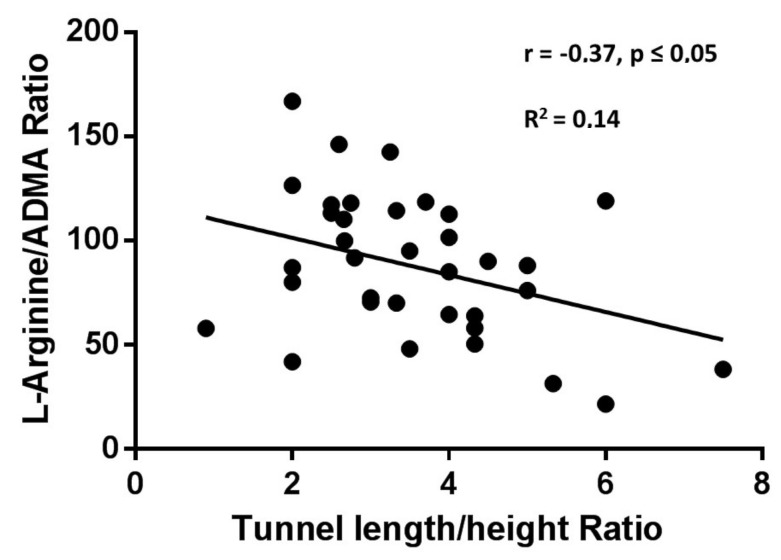
Pearson correlation and linear regression model between L-Arg/ADMA ratios and tunnel length/height ratios in patients carrying a patent foramen ovale (PFO) defect.

Figure 3 shows a negative linear correlation between the L-Arg/ADMA ratio and the tunnel-like valve length/height ratio in patients carrying a PFO defect (*p* < 0.05; r = −0.37; R^2^ = 0.14).

**Figure 4:**
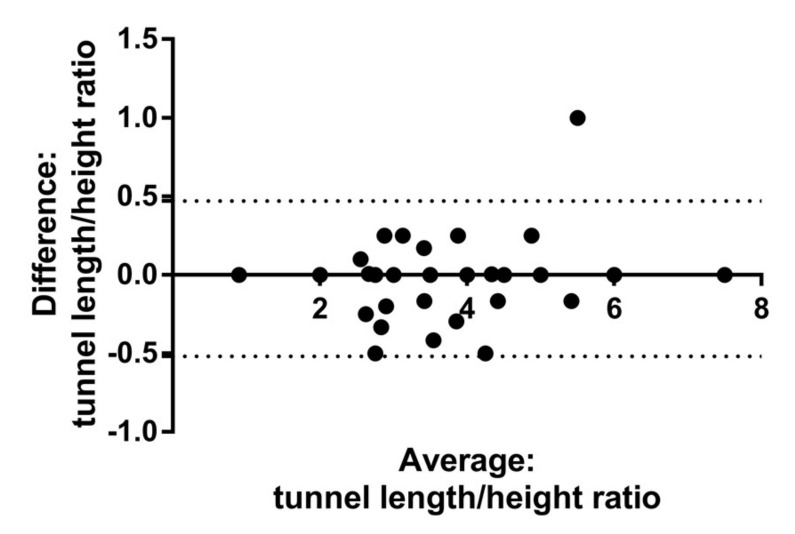
Bland–Altman analysis showing a mean bias of −0.023, with 95% limits of agreement between −0.52 and 0.47.

Inter-observer variability of tunnel length/height ratio assessed by Bland–Altman analysis revealed a mean bias of −0.023, with 95% limits of agreement of −0.52 to 0.47 (Figure 4).

### 3.5. Distribution of MTHFR Genetic Variants and IAS Phenotype

**Figure 5:**
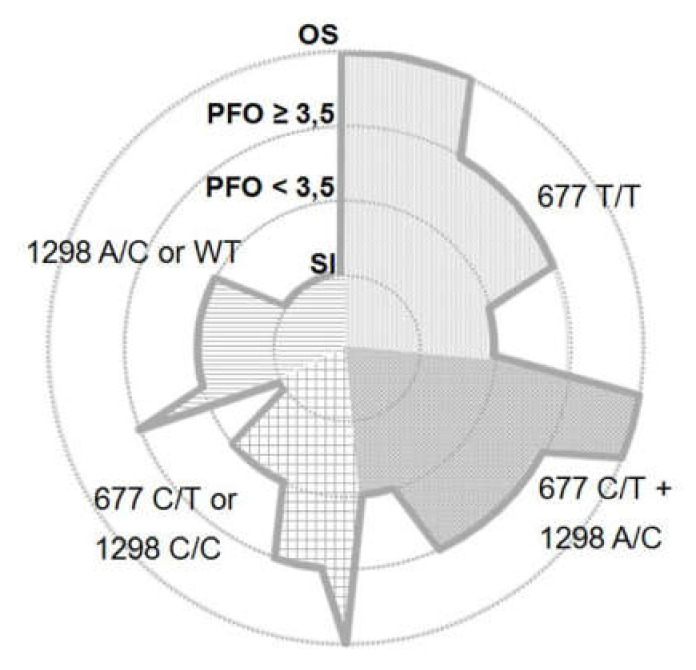
Schematic diagram illustrating the distribution of interatrial septum (IAS) phenotypes (concentric circles) with respect to the MTHFR genotypes among all patients. Subjects carrying either the 677 C/T or the 1298 C/C MTHFR genotype as well as 1298 A/C or WT genotype (mutations with similar residual enzymatic activity, respectively) were grouped together for clarity. SI = septum integrum; PFO = patent foramen ovale with tunnel length/height value below or above 3.48 (± 1.43 SD); OS = ostium secundum.

According to the IAS morphology and MTHFR genotype, the following distribution was observed (Figure 5): Among patients with an OS defect (*n* = 9), 5 were carrying the 677 T/T mutation, 3 were carrying the 677 C/T + 1298 A/C double heterozygous mutation, and 1 patient was carrying the 677 C/T heterozygous genotype.

PFO carriers were sub-grouped according to tunnel length/height ratio above or below the average value of 3.5. Of the PFO subjects with a tunnel length/height ratio above 3.5 (*n* = 18), 7 patients carried the 677 T/T mutation, 7 patients carried the 677 C/T + 1298 A/C double heterozygous mutation, 3 patients carried the 677 C/T heterozygous genotype, and 1 patient carried the 1298 A/C genotype. Among PFO subjects with tunnel length/height ratio below 3.5 (*n* = 20), 4 patients carried the 677 T/T mutation, 4 patients carried the 677 C/T + 1298 A/C double heterozygous mutation, 1 patient carried the 677 C/T heterozygous genotype, 4 patients carried the 1298 C/C homozygous genotype, and 7 patients carried the 1298 A/C MTHFR genotype. All patients with SI (*n* = 10) carried either the 1298 A/C (*n* = 4) variant or the wild-type (*n* = 4) MTHFR enzyme, except for 1 patient carrying the 677 C/T heterozygosis and another one carrying the 1298 C/C homozygosis.

## 4. Discussion

Genetic defects in MTHFR have been reportedly coupled to hyperhomocysteinemia, a marker for atherosclerosis, cardiovascular events, and microangiopathy risk [21]. Undeniably, methyl overload and disorders in the folate cycle subsequent to MTHFR mutations disturb the synthesis/function of multiple factors involved in cell regulation: In endothelium, the impaired availability of substrates (as L-Arg) and co-factors (such as tetrahydrobiopterine, BH4) of the nitric oxide (NO) synthase (eNOS) reduces production of NO, the most reliable indicator of endothelial function. We investigated whether abnormal activity of MTHFR may impair the endothelium-driven development/repair of the interatrial septum, with the hypothesis that the concomitant occurrence of these conditions may represent a stroke predisposition. In this scenario, the significance of the L-Arg/ADMA ratio as an indirect marker of endothelial function may acquire clinical importance for its potential use as both a diagnostic tool and a decision aid for therapeutic strategies.

### 4.1. Correlation between the Severity of MTHFR Activity and the Degree of Endothelial Dysfunction

Not surprisingly, higher levels of homocysteine were found in patients with the most impaired MTHFR variants; concomitantly, the L-Arg/ADMA ratio decreased proportionally to the severity of the MTHFR mutation. Several interrelated mechanisms support the idea that MTHFR-mediated disruption in the folate cycle may trigger endothelial dysfunction: Under methylic surcharge and methionine deficiency, L-Arg may be directly converted to ADMA [29], a powerful endogenous inhibitor of eNOS; moreover, homocysteine-dependent downregulation of dimethylarginine dimethyl-aminohydrolase (DDAH) results in increased ADMA levels and endothelial dysfunction [30,31], as observed in patients exposed to methionine loading tests [32]. In addition, low levels of both BH4 and NO, with subsequent endothelial dysfunction, have been observed in patients with hyperhomocysteinemia [33]. Even if easy to be suggested from a molecular perspective, the clinical demonstration of a pathogenic relationship between MTHFR mutations and ADMA still remains an unexplored field [34,35]. On this basis, the present findings represent the first attempt to demonstrate an existing relationship between MTHFR activity and the L-Arg/ADMA ratio, therefore supporting the hypothesis that MTHFR mutations influence endothelial function. Interestingly, in our study, the L-Arg/ADMA ratio behaves as a more sensitive indicator of a folate cycle disruption with respect to homocysteine levels. This last observation further reinforces the proposed idea that the L-Arg/ADMA ratio may serve as a handful marker of endothelial function, whose values may contribute to better characterize specific patient subgroups.

### 4.2. Correlation between the Degree of Endothelial Dysfunction and the IAS Phenotype

Based on the aforementioned considerations—along with its role as an independent marker of ischemic stroke [36], cardiovascular events [37], risk factor for microangiopathy-related cerebral damage [38], and silent brain infarction [39]—the L-Arg/ADMA ratio (as a surrogate of endothelial dysfunction) might be proposed as an indicator of IAS defects. In accordance with Ozdemir et al. [26], we observed that, with respect to patients with SI, levels of homocysteine were proportionally higher in patients with PFO or OS phenotypes. In parallel, the L-Arg/ADMA ratio progressively declined among a spectrum encompassing SI, PFO with shorter and larger tunnel, PFO with longer and tighter tunnel, and complete OS defects. The idea that MTHFR-mediated disorders in the folate cycle trigger endothelial dysfunction, and that this condition may in turn influence the IAS phenotype, grounds on several clinic, translational, and basic research studies: An appropriate L-Arg/ADMA ratio, indicative of a physiological NO production, is required for the proper post-natal cardiomyocyte proliferation and differentiation [40], suggesting that a fully performing eNOS is mandatory for postnatal heart development [41]. In line with this, congenital atrial septal defects have been observed in eNOS-deficient mice [42]; interestingly, impaired NO production subsequent to reduced BH4 bioavailability has been reported in mesenteric vessels from MTHFR deficient mice [43]. The importance of the C677T MTHFR mutation to promote neural tube defects is also well recognized [44,45]; similarly, the preventative effect of low-dose folate administration on stroke onset has been repeatedly confirmed [46]; these findings are consistent with the higher plasma levels of homocysteine found in PFO carriers [26]. Taken together, all these ideas contribute to the support of the possibility that MTHFR-related disorders might account for IAS defects in humans [47,48].

The correlation between features of the PFO tunnel-like valve and the L-Arg/ADMA ratio might help to reconcile current controversies concerning tunnel size and risk of cryptogenic stroke [10,11]. According to our findings, the hypothetical major contribution of larger tunnels to paradoxical embolism might be counterbalanced by the more severe endothelial dysfunction in PFO with tighter and longer tunnels. Consistent with this hypothesis, the L-Arg/ADMA ratio was similar in patients carrying either an OS or PFO defect.

### 4.3. Relationship between the Severity of MTHFR Activity, the Characteristics of IAS Defects, and Cryptogenic Strokes

Our findings strongly suggest that the most severe septum defects are found in patients carrying high-dysfunctional MTHFR variants. The relationship found between the L-Arg/ADMA ratio and the PFO tunnel morphology might partially explain why PFO prevalence decreases with age, whereas its size increases [49]; this idea is consistent with the observation that reduced MTHFR activity contributes to impair survival and function of circulating endothelial progenitor cells [50], whose inefficiency is important on stroke onset [51]. Interestingly, ADMA levels are increased in subjects reporting migraines with aura [52], and the L-Arg/ADMA ratio is accepted as an independent predictor of mortality [37].

The lack of specificity in current classification confines cryptogenic strokes to an exclusion diagnosis, wherein multiple pathogenic factors coexist. While paradoxical embolism cannot explain strokes occurring in patients with no interatrial abnormality, or carrying a septal aneurysm not associated with a right-to-left shunt, the presence of endothelial dysfunction may help to unravel a potentially unrecognized contributor to cryptogenic stroke. Consistent with our hypothesis, lower values of flow-mediated dilation (indicating endothelial dysfunction) have been proposed as an independent risk factor for strokes, irrespective of PFO presence [53].

## 5. Limitations of the Study

The following limitations should be taken into account when evaluating the overall message of our study: First of all, the narrow number of patients evaluated does not allow an authoritative indication of a direct cause–effect relationship between MTHFR genotype, IAS defects, and cryptogenic strokes. In this respect, the demographic characteristics of subjects enrolled might represent an important drawback: For example, although PFO and MTHFR-inherited thrombophilia share roughly the same prevalence worldwide [20,54,55], ischemic strokes have been related to PFO with larger tunnels and a low frequency of MTHFR mutations in a black population; on the other hand, PFO with tighter tunnels and a high frequency of MTHFR mutations have been documented in Hispanic patients undergoing strokes [56,57]. More inclusive studies will hopefully help to ascertain the specific risk in a sub-population of patients.

One second point is related to the use of the L-Arg/ADMA ratio to indicate endothelial dysfunction. Although strongly suggestive of a relationship between impaired MTHFR activity and abnormal endothelial function, the L-Arg/ADMA ratio does not give information on the intracellular content and activity of key molecules or signaling cascades. One possibility to corroborate the link between the L-Arg/ADMA ratio and the degree of endothelial performance in patients with cryptogenic stroke could come by the characterization of pro-angiogenic molecular signaling from circulating Endothelial Progenitor Cells (EPCs). It has been proven that the level of circulating EPCs is an independent predictor of the prognosis for patients with an acute ischemic stroke, and that circulating EPCs are significantly impaired in patients with cerebro-cardiovascular diseases with respect to control subjects. Since EPCs can differentiate into endothelial cells, replacing or directly integrating with the damaged endothelial layer, it is likely that any alteration in their expression pattern of eNOS or caspases might reflect the impaired activity of mature endothelial cells. Unfortunately, because of the retrospective nature of our study, these experiments could not be carried out at present. Nevertheless, and consistent with literature data, impaired eNOS protein expression and NO production with concomitant increased ROS production and NF-kB activation were observed in human endothelial cells incubated in vitro under high homocysteine concentrations. If confirmed, these observations—too preliminary to be shown at present—might provide further support to our idea of a tight link between folate-related endothelial function and unbalanced L-Arg/homocysteine levels.

Moreover, several other possibilities exist: For example, the consequences of MTHFR defects might extend to abnormal function of other vascular cells types, such as smooth muscle cells. It is overly accepted that vascular cell proliferation may play an important role in the pathogenesis of cerebral vasospasm, and that both hyperhomocysteinemia and folate deficiency may influence key processes such as methylation and global gene expression patterns in smooth muscle cells. Finally, endothelial trans-differentiation process towards a more contractile phenotype, mostly referred as endothelial to mesenchymal transition (EndMT) could be jeopardized. In EndMT, endothelial cells adopt a mesenchymal phenotype displaying typical mesenchymal cell morphology and functions, including the acquisition of fiber deposition (myofibroblast) and contractile properties (smooth muscle cell).

In summary, considering the increasing recognition on the contribution that MTHFR plays in a myriad of physiological processes involved in the differentiation from endothelial progenitor cells->endothelial cells->vascular smooth cells or myofibroblasts, the lack of an established causative effect might represent the most significant limitation of this paper. Notwithstanding, findings provided here open a standpoint from which conceive novel perspectives. Altogether, the multifaceted and still largely incomplete knowledge on mechanisms underlying cryptogenic stroke highlight the critical importance of continued studies in this field.

## 6. Conclusions

From a clinical perspective, our results may contribute to clarify the current scenario of diagnostic and therapeutic uncertainty in patients with cryptogenic strokes. If validated, the L-Arg/ADMA ratio may represent a reliable marker of stroke susceptibility in carriers of IAS abnormalities implying that, in the near future, therapeutic strategies targeting endothelial dysfunction—in addition to antiplatelet and anticoagulant therapies—may reveal their importance in stroke primary prevention. Moreover, our findings may help to identify subgroups of subjects that would take full advantage from PFO surgical closure over medical therapy, as well as subjects that would instead obtain the maximal beneficial effects from folate administration to reduce stroke incidence [46,58].

## Figures and Tables

**Table 1:** Clinical characteristics of patients according to septum phenotype.

	SEPTUM INTEGRUM	PATENT FORAMEN OVALE	OSTIUM SECUNDUM
PATIENTS (N)	10	38	9
**Age** (year)	37 ± 10	42 ± 11	40 ± 17
**Fibrinogen** (mg/dL)	264 ± 36	249 ± 41	239 ± 43
**D-dimers** (ng/dL)	488 ± 421	463 ± 370	387 ± 122
**P-Hcy** (µmol(L)	7.2 ± 3.6	12.1 ± 7.6 *	12.7 ± 3.2 *
**Folates** (ng/mL)	5.0 ± 4.2	7.7 ± 4.5	17.7 ± 9.9 *
**B12 Vit** (pg/mL)	438.4 ± 82.3	417.9 ± 166.1	341 ± 104.3
**Creatinine** (mg/dL)	0.65 ± 0.13	0.75 ± 0.13	0.70 ± 0.19
**eGFR** (mL/min)	103 ± 15	102 ±13	108 ± 19
**Glucose** (mg/dL)	96 ± 13.5	88.6 ± 9.5	76 ± 11.2
**Cholesterol** (mg/dL)	209 ± 27	182 ± 26	201 ± 12
**HDL** (mg/dL)	53 ± 9	59 ± 14	55 ± 5
**LDL** (mg/dL)	125 ± 45	103 ± 21	116 ± 12
**Triglicerides** (mg/dL)	110 ± 34.5	75 ± 24.2	60 ± 18.2
**Systolic BP** (mmHg)	118 ± 5.2	121 ± 4.78	119 ± 12.2
**Diastolic BP** (mmHg)	74 ± 9.5	75 ± 3.3	70 ± 9.4

* *p* < 0.05 *vs.* respective values in the SI group.

**Table 2:** Clinical characteristics of patients according to MTHFR genotype.

	677 T/T	677 C/T + 1298 A/C	677 C/T	1298 C/C	1298 A/C + WT
PATIENTS (N)	16	14	6	5	16
**Age** (year)	39 ± 12	40 ± 9	46 ± 18	40 ± 16	42 ± 9
**Fibrinogen** (mg/dL)	225 ± 24	268 ± 39	260 ± 37	264 ± 65	263 ± 44
**D-dimers** (ng/dL)	356 ± 153	466 ± 438	462 ± 298	653 ± 585	349 ± 322
**P-Hcy** (µmol/L)	16.3 ± 5.8 *	9.8 ± 2.9	8.0 ± 2.1	7.9 ± 1.1	9.1 ± 2.9
**Folates** (ng/mL)	10.6 ± 4.4	7.9 ± 2.7	7.9 ± 5.6	11.4 ± 4.1	6.6 ± 3.3
**B12 Vit.** (pg/mL)	383.8 ± 150.3	376.3 ± 88.9	385.5 ± 112.5	541 ± 202.5	440.9 ± 199.4
**Creatinine** (mg/dL)	0.60 ± 0.06	0.79 ± 0.18	0.73 ± 0.21	0.72 ± 0.09	0.77 ± 0.09
**eGFR** (mL/min)	110.5 ± 6.4	102 ± 2.8	92 ± 26.8	109.5 ± 10.6	101.75 ± 12.3
**Glucose** (mg/dL)	87 ± 8.04	107.5 ± 12.02	86 ± 4.24	93.5 ± 23.33	95 ± 10.61
**Cholesterol** (mg/dL)	181 ± 24.3	172 ± 45.9	195 ± 30.4	182 ± 17.7	188 ± 54.1
**HDL** (mg/dL)	56 ± 7.5	53 ± 2.8	83 ± 4.9	46 ± 5.6	51 ± 16.2
**LDL** (mg/dL)	110 ± 28.2	101 ± 38.9	101 ± 24.4	112 ± 7.8	113 ± 40.9
**Triglicerides** (mg/dL)	78 ± 27.5	91 ± 49.5	54 ± 8.5	83 ± 21.2	69 ± 16.3
**Systolic BP** (mmHg)	117 ± 6.2	120 ± 7.8	122 ± 2.2	118 ± 5.6	119 ± 8.1
**Diastolic BP** (mmHg)	76 ± 5.5	78 ± 4.3	79 +6.4	79 ± 2.5	76 ± 10.2

* *p* < 0.01 *vs.* respective value in all other MTHFR subgroups.

## Supplementary Materials

The following are available online at https://www.mdpi.com/2218-273X/10/6/861/s1, Figure S1: L-Arg/ADMA ratios across healthy volunteers, cryptogenic stroke patients, and acute myocardial infarction.

## Abbreviations

MTHFR	methylene tetrahydrofolate reductase
PFO	patent foramen ovale
IAS	interatrial septum
OS	ostium secundum
ADMA	asymmetric dimethylarginine
