# Supplementary material for: Endothelial Dysfunction May Link Interatrial Septal Abnormalities and MTHFR-Inherited Defects to Cryptogenic Stroke Predisposition
Source: Biomolecules. 2020 Jun 4;10(6):861. doi: 10.3390/biom10060861 (PMC7355772; doi:10.3390/biom10060861)
Supplement: Supplementary file 1 [file biomolecules-10-00861-s001.pdf]

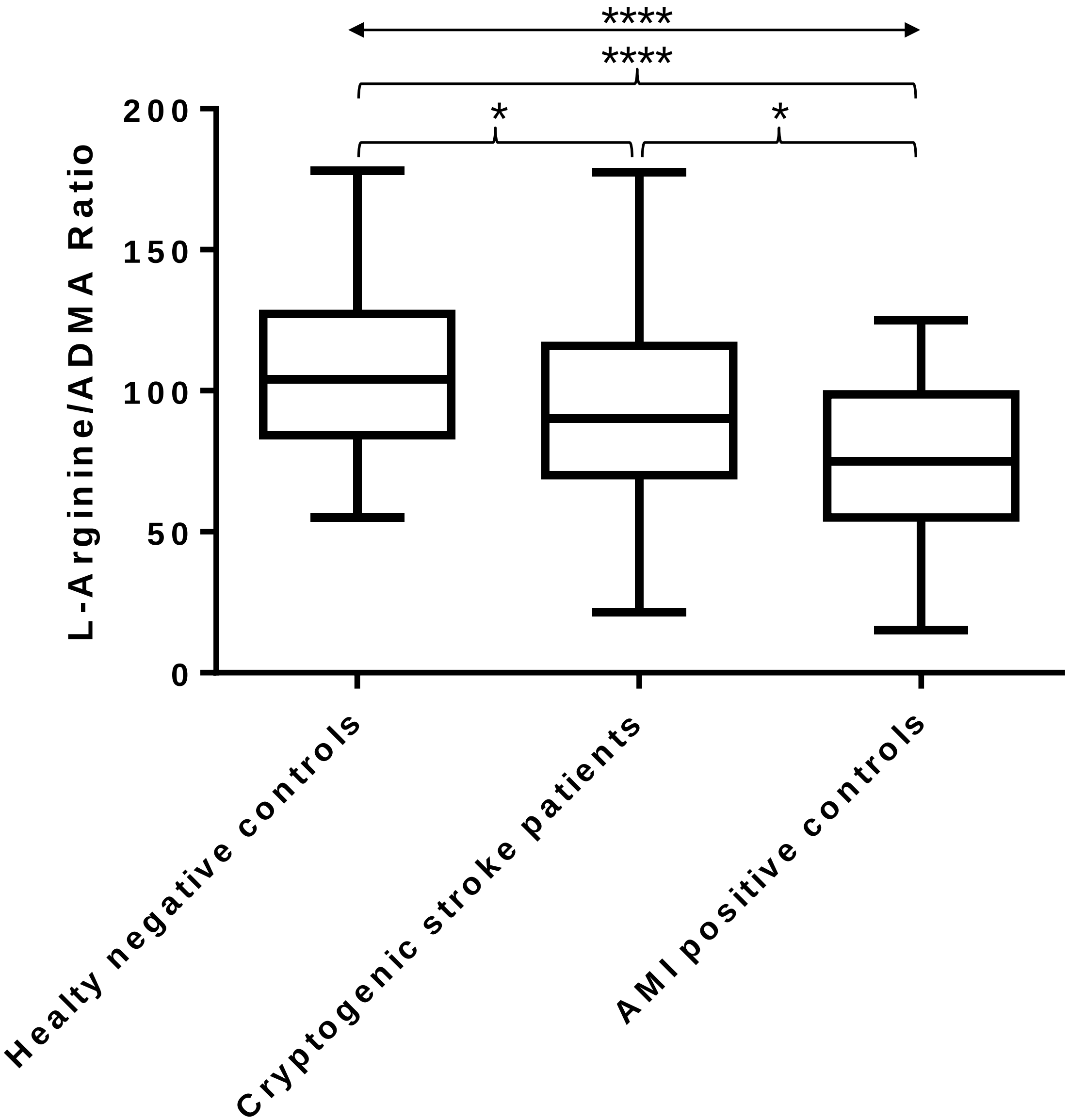

Suppl. Figure 1. L-Arg/ADMA ratios across healthy volunteers (negative controls, n=48), cryptogenic stroke patients (n=57) and acute myocardial infarction patients (AMI, positive controls, n=48). Box plots indicate the median, maximum and minimum value. P value across groups (double arrow line) was calculated by Kruskall-Wallis non parametric test of one-way ANOVA data. P value between groups (curly brackets) was calculated by Dunn's multiple correction.
* $p \leq 0.05$; **** $p \leq 0.0001$
